# Guillain‐Barré syndrome following COVID‐19 vaccination: An updated systematic review of cases

**DOI:** 10.1002/ccr3.7456

**Published:** 2023-06-07

**Authors:** Nour Shaheen, Abdelraouf Ramadan, Abdulqadir J. Nashwan, Ahmed Shaheen, Shahzaib Ahmad, Karam R. Motawea, Salaheldin Mohamed, Rahma Sameh Mohamed, Sarya Swed, Hani Aiash

**Affiliations:** ^1^ Faculty of Medicine Alexandria University Alexandria Egypt; ^2^ Department of Neurosurgery and Brain Repair University of South Florida Tampa Florida USA; ^3^ Faculty of Medicine Helwan University Cairo Egypt; ^4^ Nursing Department Hamad Medical Corporation Doha Qatar; ^5^ King Edward Medical College: King Edward Medical University Lahore Pakistan; ^6^ Faculty of Medicine Benha University Benha Egypt; ^7^ Faculty of Medicine Aleppo University Aleppo Syria; ^8^ Cardiovascular Perfusion Department Upstate Medical University Syracuse New York USA

**Keywords:** COVID‐19, COVID‐19 vaccines, Guillain‐Barré syndrome (GBS), SARS‐CoV‐2

## Abstract

**Key Clinical Message:**

Guillain‐Barré syndrome (GBS) is a rare but possible complication that may occur after COVID‐19 vaccination. In this systematic review, we found that GBS presented in patients with an average age of 58. The average time for symptoms to appear was 14.4 days. Health care providers should be aware of this potential complication.

**Abstract:**

Most instances of Guillain‐Barré syndrome (GBS) are caused by immunological stimulation and are discovered after vaccinations for tetanus toxoid, oral polio, and swine influenza. In this systematic study, we investigated at GBS cases that were reported after receiving the COVID‐19 vaccination. Based on PRISMA guidelines, we searched five databases (PubMed, Google Scholar, Ovid, Web of Science, and Scopus databases) for studies on COVID‐19 vaccination and GBS on August 7, 2021. To conduct our analysis, we divided the GBS variants into two groups, acute inflammatory demyelinating polyneuropathy and non‐acute inflammatory demyelinating polyneuropathy (AIDP and non‐AIDP), and compared the two groups with mEGOS and other clinical presentation In this systematic review, 29 cases were included in 14 studies. Ten cases belonged to the AIDP variant, 17 were non‐AIDP (one case had the MFS variant, one AMAN variant, and 15 cases had the BFP variant), and the two remaining cases were not mentioned. Following COVID‐19 vaccination, GBS cases were, on average, 58 years of age. The average time it took for GBS symptoms to appear was 14.4 days. About 56 percent of the cases (56%) were classified as Brighton Level 1 or 2, which defines the highest level of diagnostic certainty for patients with GBS. This systematic review reports 29 cases of GBS following COVID‐19 vaccination, particularly those following the AstraZeneca/Oxford vaccine. Further research is needed to assess all COVID‐19 vaccines' side effects, including GBS.

## Graphical abstract

Prisma Flow diagram of the studies included in the systematic review;GBS following COVID‐19 vaccination.

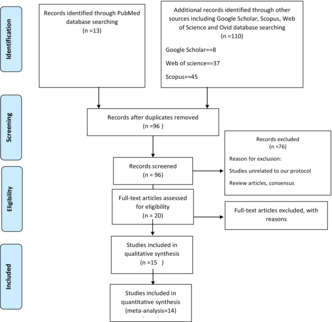

## 1 INTRODUCTION

More than 267 million individuals have been infected with COVID‐19, which is spurred on by the severe acute respiratory syndrome coronavirus‐2 (SARS‐CoV‐2) and has killed 4.8 million people globally.^1^, ^2^ The development of vaccines has been ongoing since the beginning of the pandemic. The vaccines became available worldwide in 2021, which was an early milestone that helped relieve the COVID‐19 pandemic. Vaccination was able to slow down the spread of infection, allowing hospitals to heal from an influx of patients during peak incidence.^3^ About half of the world's population (55.5%) has received a dose of the COVID‐19 vaccine at least once, 8.35 billion doses have been administered worldwide, and 30.58 million doses are administered daily.^4^, ^5^, ^6^

Although vaccinations such as those from Pfizer and AstraZeneca are effective and safe, some recipients have reported side effects.^7^, ^8^ As a result of taking the vaccine, mild symptoms such as soreness, headaches, fatigue, chills, joint pain, nausea, muscle spasms, sweating, and dizziness may occur.^9^, ^10^, ^11^ A few cases of Guillain‐Barré Syndrome (GBS) have been reported following vaccination.^12^, ^13^ GBS is a rare immune‐mediated polyradiculoneuropathy in which the immune system attacks peripheral nerves following infection with a virus or bacteria. GBS has no clearly defined cause, but it may occur after infection with a virus or bacteria. In rare cases, GBS can also be preceded by vaccination.^8^

The small risk of GBS associated with the swine influenza vaccine used in 1976–77 suggests this possible causal association. Also, older formulations of the rabies vaccine were found to increase the risk of GBS. It has been suggested that oral polio vaccines and tetanus toxoid‐containing vaccines might cause GBS. Recently, the US Vaccine Adverse Events Reporting System reported an association between GBS and the quadrivalent meningococcal vaccine (MCV4).^14^, ^15^

GBS is an autoimmune disorder in which the body's immune system attacks the peripheral nerves. This results in the inflammation and the damage of the nerves which can lead to various affects throughout the body. This can range from numbness to paralysis.^16^

We introduce in this systematic review 13 case reports and case series studies, as well as one letter describing confirmed cases of GBS after vaccination against the COVID‐19 virus.

## 2 METHODS

### 2.1 Study design

**FIGURE 1:**
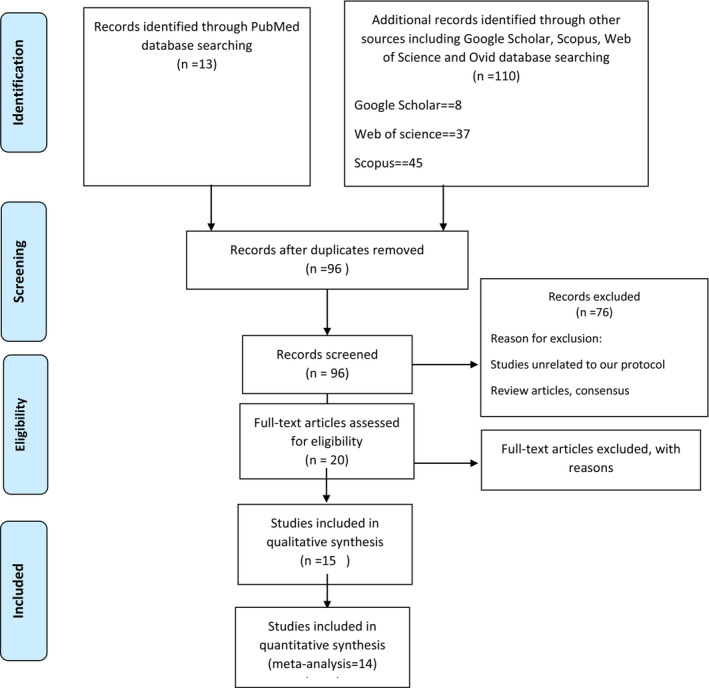
PRISMA flow diagram of the systematic review of case reports.

We conducted a thorough literature review in August 2021 using the terms ((“Guillain‐Barre Syndrome”[Mesh]) OR “Miller Fisher Syndrome”[Mesh]) AND “COVID‐19 Vaccines”[Mesh], TITLE‐ABS‐KEY (Guillain AND barre AND (COVID‐19 AND vaccine*)). We searched PubMed, Google Scholar, Ovid, Web of Science, and Scopus databases for identifying case series and case reports published on August 7, 2021, for COVID‐19; Two reviewers separately searched to find the studies that matched the search terms. Studies describing the cases of Guillain Barré Syndrome following COVID‐19 vaccination (Figure 1); in addition, the analysis did not include review articles or consensus statements. The Preferred Reporting Items for Systematic Reviews and Meta‐Analyses (PRISMA) were used to illustrate inclusions and exclusions [20]. We discovered 113 studies from PubMed, Google Scholar, Ovid, Web of Science, and Scopus based on our search parameters. 95 full‐text publications were evaluated after the exclusion of duplicate studies, studies with incomplete clinical data, review articles, and papers irrelevant to our research purpose. Accordingly, 14 studies of COVID‐19 immunization and GBS were examined for descriptive analysis. These 14 articles were included in our evaluation since they satisfied our aforementioned inclusion criteria. (Figure‐1).

### 2.2 Inclusion criteria

The published studies' inclusion criteria included the following:

1. GBS confirmed in post‐COVID‐19 vaccine recipients by clinical manifestation and diagnostic procedures including EMG and cerebrospinal fluid (CSF) testing.

### 2.3 Exclusion criteria

The exclusion criteria for the studies include:

1. Individuals who had vaccinations and were diagnosed with a condition other than GBS, such as myopathy, toxin induced polyneuropathy, critical illness polyneuropathy (CIP), or critical illness myopathy.
2. Studies that used repeated instances in duplicate
3. Languages other than English studies.
4. Exclusion of studies without a confirming GBS diagnosis.

### 2.4 Quality assessment

The overall quality of case series and case reports has been evaluated using the JBI (Joanna Briggs Institute) Critical Appraisal Checklist for Case Reports.

### 2.5 Data acquisition

For our analysis, we took the following information from the chosen studies: research type, date of publication, country of case origin, age, gender, clinical presentation of GBS and its variations, such as paraparesis/quadriparesis and cranial nerve deficits, diagnostic tests for SARS‐CoV‐2 infection, such as RT‐PCR nasopharyngeal, and delay between COVID‐19 immunization and early signs of GBS.

### 2.6 Data analysis

For all patients throughout the 14 case reports and series, pooled descriptive analyses were performed to compare the differences between AIDP and Other GBS variations, the two primary categories of GBS variants (comprising of AMSAN, AMAN, BFP, MFS, Polyneuritis cranialis). Using the chi‐square test for categorical covariates and the t‐test for continuous covariates, we evaluated the differences between two groups for the variables. Additionally, a sub‐analysis of the variations in frequencies and proportions across three groups made up of AIDP and non‐AIDP individuals was carried out. The Chi‐square test was used in other statistics using IBM SPSS Statistics version 25.

## 3 RESULTS

The study included 29 cases from 14 studies. The GBS variants were divided into two categories (AIDP and non‐AIDP). Consequently, 10 of the 27 cases were AIDP, 17 non‐AIDP, and two cases lacked data regarding the GBS variant type, so they were not included in the analysis but discussed. We found no statistically significant difference in age between the two groups (*p* = 0.920). There were 16 males and 11 females in the study, and no differences were observed between the two groups regarding gender (*p* = 0.373). We detected the difference between the two groups regarding the type of the vaccine. None of the three vaccines showed a significant difference between the two groups concerning the incidence of GBS (Table 1). One patient was previously tested positive for SARS‐COV‐2 in the non‐AIDP group, and all patients in the AIDP group were negative (*p* = 0.729). one patient in each group was admitted to ICU (*p* = 0.348). We observed no statistical difference regarding the time from vaccination to onset of GBS between the two groups (*p* = 0.420) (Table 2). Protein values ranges were (54–900) and (75–722) in AIDP and non‐AIDP, respectively, (*p* = 0.392). mEGOS score means and standard deviations were (6 ± 5.51) and (10.38 ± 1.06) in AIDP and non‐AIDP, respectively; the *p*‐value was significant (*p* = 0.050), indicating a significant association between non‐AIDP and increasing mEGOS score compared to AIDP. Albumino‐cytological dissociation was present in 2 patients in the AIDP group and present in 15 patients in the non‐AIDP group; *p*‐value was significant (*p* = 0.000), indicating a significant association between non‐AIDP and the presence of Albumino‐cytological dissociation compared to AIDP. One patient was mechanically ventilated in the AIDP group, and five patients were mechanically ventilated in the non‐AIDP group; the *p*‐value was significant (*p* = 0.002), and this shows a significant association between the non‐AIDP group and increasing mechanical ventilation compared to the AIDP group (Table 1).

**TABLE 1:** Demographics of the included patients and their outcomes.

Characteristics	AIDP	Non‐AIDP	*p*‐Value
Number of patients	9	18	
Age, years (Mean ± SD)	58.6 ± 11.7	58.1 ± 13.2	0.920
Gender			0.373
Male	7	9	
Female	2	9	
(SARS‐COV‐2) status			0.729
Positive	0	1	
Negative	9	17	
Type of the vaccine			
AstraZeneca	8	17	0.146
Johnson &Johnson	0	1	0.729
Pfizer	1	0	0.250
ICU admission	1	1	0.348
Time from vaccination to onset of GBS (Mean ± SD)			
Protein (range) (g/dL)			
mGEOS score (Mean ± SD)	12.9 ± 3.6	14.8 ± 8.7	0.420
Albumino‐cytological dissociation			
Present	(54–900)	(75–722)	0.392
Absent	6 ± 5.51	10.38 ± 1.06	0.050
Mechanical ventilation			0.000
	3	14	
	6	1	
	1	5	0.002

*Note:* p < 0.05 is considered significant.

Abbreviations: SD, standard deviation; mGEOS, Modified Erasmus GBS Outcome Score.

**TABLE 2:** Characteristics of the included patients and their outcomes.

Characteristics	AIDP	Non‐AIDP	*p*‐Value
Bilateral facial weakness.	4	10	0.035
General muscle weakness	6	7	0.000
Upper limb weakness	5	7	0.000
Lower limb weakness	7	10	0.000
Paraesthesia	4	11	0.038
Quadriparesis	3	2	0.001
Paraplegia	1	0	0.003
Quadriplegia	0	3	0.004
Ascending paralysis	2	1	0.005
Numbness	7	13	0.000
Diarrhea	1	0	0.001
Headache	1	3	0.026
Bell's palsy	3	1	0.003
Back pain	0	8	0.003
Visual disturbances	0	1	0.002
Diplopia	0	2	0.006
Dysphagia	2	4	0.012

Brighton criteria level of diagnostic certainty, MRC grade at the upper and lower limb, and predicted probability to walk unaided after 4 weeks, 3 months, and 6 months are classified in the two groups in Table 3.

**TABLE. 3:** Study origin, types, demographics, and GBS variants.

S. No.	Author	Country	Type of study	No. of patient	Mean age	Gender	GBS variant	Type of COVID‐19 vaccine
1	(Marquez Loza et al. 2021)^17^	USA	Case report	1	60	Female	MFS	Johnson &Johnson
2	(Waheed et al. 2021)^18^	USA	Case report	1	81	Female		Pfizer
3	(James et al. n.d.)^19^	India	Case series	3	60	2 males	AIDP	(AstraZeneca/oxford)
						female	AIDP	
							AIDP	
4	(R. A et al. 2021)^20^	Qatar	Case report	1	73	Male	AIDP	Pfizer
5	(Tanveer Hasan et al. 2021)^21^	UK	Case report	1	62	Female	AIDP	(AstraZeneca/oxford)
6	(J 2021)^22^	Austria	Case report	1	32	Male	AIDP	
7	(Nasuelli et al. n.d.)^23^	Italy	Case report	1	59	Male	AIDP	(AstraZeneca/oxford)
8	(Allen et al. 2021)^24^	UK	Case report	4	46.5	4 males	1AIDP	(AstraZeneca/oxford)
							1 BFP	
							1 BFP	
							1 AMAN	
9	(Azam, Khalil, and Taha 2021)^25^	UK	Case report	1	67	Male	AIDP	the first dose of the (AstraZeneca/oxford)
10	(Patel et al. 2021)^26^	UK	Case report	1	37	Male		the first dose of (AstraZeneca/oxford)
11	(Finsterer 2021)^27^	Austria	Case report	1	69	Female	AIDP	The first dose of (AstraZeneca/oxford)
12	(Maramattom et al. 2021)^28^	India	Case series	7	62.7	6 females	BFP	first dose of the ChAdOx1‐S vaccine
						1 male		
13	(Bonifacio et al. 2021)^29^	UK	Letter	5	56.8	4 men	BFP	the first dose of (AstraZeneca/oxford)
						1 female		
14	(I. A et al. 2021)^30^	Italy	Case report	1	62	Male	BFP	First dose of (AstraZeneca/oxford
15	([Anonymous] 2021)^31^	USA	Letter	100				Johnson & Johnson

We found a statistically significant association between AIDP and incidence of general muscle weakness, upper limb weakness, lower limb weakness, quadriparesis, paraplegia, ascending paralysis, numbness, diarrhea, Bell's palsy, and dysphagia (Table 4). On the other hand, we found a significant difference between non‐AIDP and incidence of bilateral facial weakness, paresthesia, quadriplegia, headache, visual disturbances, back pain, and diplopia (Table 4).

**TABLE 4:** Descriptive characteristics of cases with Guillain barre syndrome following COVID‐19 vaccination.

		AIDP, *N* = 8	Non AIDP, *N* = 19	Total, *N* = 27
Age		58.6 ± 11.8	57.4 ± 13.2	57.8 ± 12.5
Gender	Male	6	10	16
	Female	2	9	11
Total		8	19	27
mEGOS score (Mean ± SD)		6 ± 5.51	10.38 ± 1.06	8.3 ± 3.6
Brighton criteria		AIDP	Non AIDP	Total
Level 1		1	6	3
Level 2		1	5	12
Level 3		2	7	1
Level 4		1	0	1
The duration between CoV vaccination and GBS onset		12.50 ± 3.66	14.89 ± 8.45	14.41 ± 7.23
Albumino‐cytological	Absent	6	1	7
dissociation				
	present	2	15	17
Total		8	16	24
MRC at upper limb		4.13 ± 1.13	2.67 ± 1.32	3.44 ± 1.42
MRC at lower limb		3.38 ± 1.4	2 ± 1.5	2.72 ± 1.56
Clinical presentation				
numbness	Present	6	14	20
	Absent	2	5	7
Upper limb weakness	Present	5	7	12
	Absent	2	11	13
Lower limb weakness	Present	7	10	17
	Absent	0	8	8
Bell's palsy	Present	3	1	4
	Absent	2	15	17
Cranial nerve VII palsy	Present	1	8	9

## 4 DISCUSSION

There has been extensive research into Guillain‐Barré syndrome associated with various vaccines available to understand its association with the disease.^32^ As a result of our study, we found 14 research articles reporting 29 cases of vaccination against SARS‐CoV‐2 infection resulting in GBS syndrome. A total of 14 studies were included, of which 13 were case reports and series and one letter. The mean age of the cases that got GBS after receiving COVID‐19 vaccination was 58 years old. The details of the studies, including the type of study, country, number of patients, mean age, GBS variant, and type of vaccine, have been tabulated in Table 3. There were two cases from the United States, two from Italy, 12 from the United Kingdom, 10 from India, two from Austria, and one from Qatar (Table 3). An included letter reported 100 cases of GBS from the USA after receiving Johnson & Johnson's first dose.^31^ Of the 29 cases, 44.8% were female and 55.2% were male. Out of the 29 cases, 26 received a first dose of AstraZeneca/Oxford vaccine, one received Johnson & Johnson vaccine, and two received Pfizer vaccine. There are several subtypes of GBS based on how it manifests clinically; acute inflammatory demyelinating polyradiculoneuropathy is the most prevalent kind (AIDP). Axonal forms such as acute motor axonal neuropathy (AMAN) and acute motor‐sensory axonal neuropathy are among the other categories (AMSAN). Miller Fisher syndrome is a regional form of GBS. A diagnosis of GBS is based on a combination of clinical presentation, CSF analysis (characterized by an increased protein level without pleocytosis), and electrophysiological criteria. The chronic form of GBS is known as chronic inflammatory demyelinating polyneuropathy (CIDP)^33^; accordingly, 10 patients had AIDP, one MFS, and one AMAN variants, while 15 patients had BFP variants of GBS (Table 3).

Furthermore, we utilized the Brighton criteria for the intensity of diagnosis and the mEGOS score to differentiate the certainty of categorization for GBS variations (14). This criterion is used to provide levels 1–4 of diagnostic certainty based on the patient's clinical presentation, exam results, and diagnostic tests.^14^ The Brighton Criteria of diagnostic certainty of GBS was discussed in eight out of the total studies. Seven patients reached Level 1, seven patients reached Level 2, 10 patients reached Level 3, and one patient reached Level 4 of the Brighton Criteria. (Table 5).

**TABLE 5:** Electromyographic features mEGOS score, Brighton Criteria, management, NCS findings, MRI findings.

S. No.	No. of patient	PCR Test for SARS‐CoV‐2 Negative	GBS variant	Management	The duration between CoV vaccination and GBS	Brighton criteria	Modified Erasmus GBS Outcome(mEGOS)Score at Day 7 of admission	NCS findings	MRI findings
1	1	Negative	MFS	IVIG	14 days			Not Tested	Brain Normal
									Lumbar Spine Enhancement of Cauda Equina
2	2	Negative		IVIG	14 days	3	2	Not tested	Brain Not Available
									Lumbar spine showed enhancement of Cauda Equina
3	3	Negative	AIDP	IVIG	11 Days		11	Sensorimotor axonal neuropathy	Normal
3	4	Negative	AIDP	IVIG	12 Days		11	Sensorimotor demyelinating neuropathy with secondary axonopathy.	Slightly Abnormal
3	5	Negative	AIDP	IVIG	13 Days		11	Sensorimotor demyelinating neuropathy with secondary axonopathy	Normal
4	6	Negative	Negative	IVIG, methylprednisolone	20 days	3	2	Axonopathy showed bilateral absent H reflexes in the gastrocnemius muscles consistent with early poly‐neuro radiculopathy	Intervertebral disk
5	7	Negative	Negative	IVIG, methylprednisolone	11 days	2	N\A	The nerve conduction study (NCS) was performed thereafter which showed marked, demyelinating, sensorimotor polyneuropathy.	Contrast enhancement
6	8	Negative	AIDP	IVIG	8 days	3	0	Nerve conduction studies had revealed slowed nerve conduction velocity, prolonged distal latencies, and absent F‐wave responses.	Nonspecific T2‐Hyperintensities
7	9	Negative	AIDP	IVIG	10 days	4	1	Not Available	Unremarkable
8	10	Negative	1AIDP	IVIG	11‐22 days	1	N\A	Facial nerve conduction studies (NCS) revealed normal terminal latencies bilaterally (2.92–3.85 ms) and significantly decreased compound muscle action potential amplitude responses (0.6–1.7 mV). There was no volitional motor activity and active denervation in the right orbicularis oris and oculi. Active denervation was seen in the left orbicularis oris and oculi, along with sporadic rapid firing, long‐duration polyphasic units, and drastically diminished recruitment. The upper and lower limbs' sensory and motor NCS were both normal.	Contrast enhanced Brain MRI
			1 BFP						
			1 BFP						
			1AMAN						
8	11	Negative	1 BFP	IVIG	11‐22 days	1	N\A	Borderline normal amplitude responses (3.2–3.3 mV) and normal terminal latencies (2.7–3.65 ms) were seen in facial NCS. In addition to early recruited fast‐firing polyphasic units of short duration and low amplitude, the orbicularis oculi and oris bilaterally displayed active denervation. The upper and lower limbs' sensory and motor NCS were both normal. The right ulnar nerve's minimum F‐wave latencies were 28 milliseconds, but the tibial nerves' latencies were between 49 and 50 milliseconds.	Normal
8	12	Negative	1 BFP	IVIG	11‐22 days	1	N\A	The upper and lower limbs' sensory and motor NCS were both normal. In the median nerves, the minimum F‐wave latencies ranged from 26 to 33 milliseconds.	Normal
8	13	Negative	1AMAN	IVIG	11‐22 days	1	N\A	Not performed	Enhancement of facial nerve with contrast
9	14	Negative	AIDP	IVIG	15 days	1	N\A	A nerve conduction analysis revealed uneven attenuation of upper limb motor responses to direct stimulation.	
									Enhancement of bilateral facial nerve
10	15	Negative		IVIG	21 days	2	3	The motor responses of the upper limbs following direct stimulation were patchily attenuated, according to a nerve conduction investigation.	Normal brain
									Thickened Cauda Equina
11	16	Positive	AIDP	IVIG	40 days	1	N\A	Nerve conduction studies revealed proximal neuropathy and demyelination because GBS was classified as acute, inflammatory, demyelinating polyneuropathy (AIDP)	Not available
12	17	Negative	BFP	IVIG/IMV	10 days	2	10	The symptoms of demyelinating neuropathy include delayed distal motor latencies, slowed conduction velocity, extended F waves, and no sensory nerve action potentials.	Not available
12	18	Negative	Unknown	IVIG/IMV/Plasmapheresis	14 Days	2	11	sensory and motor axonal neuropathy (Reduced compound motor action potentials, absent F waves, absent sensory nerve action potentials). MRI Brain–Normal	MRI brain–normal
12	19	Negative	Unknown	IVIG/IMV	12 Days	2	10	Neuropathy with demyelination (delayed distal motor latencies, slowing of conduction velocity, prolonged F waves, prolonged onset latencies of sensory nerve action potentials)	Normal
12	20	Negative	Unknown	IVIG/IMV	14 Days	2	11	Diffuse demyelinating neuropathy (delayed distal motor latencies, slowing of conduction velocity, prolonged F waves, prolonged onset latencies of sensory nerve action potentials)	Normal
12	21	Negative	Unknown	IVIG/IMV	11 Days	3	11	Demyelinating neuropathy (delayed distal motor latencies, slowing of conduction velocity, prolonged F waves, prolonged onset latencies of sensory nerve action potentials)	Normal
12	22	Negative	Unknown	IVIG/Plasmapheresis	12 Days	3	11	Demyelinating neuropathy (delayed distal motor latencies, slowing of conduction velocity, prolonged F waves, prolonged onset latencies of sensory nerve action potentials)	Not Available
12	23	Negative	Unknown	IVIG/IMV	13 Days	2	11	Demyelinating neuropathy (longer F waves, slower conduction velocity, longer F waves, and longer onset latencies of sensory nerve action potentials are all signs of delayed distal motor latencies.)	Not Available
13	24	Negative	BFP	IVIG	7 days	3	N\A	Sensory NCS: UL: absent SNAPs LL: normal Motor NCS: UL and LL: Prolonged DMLs, and F‐wave latencies Slow CV Dispersed CMAPs and CB	Normal except for Bilateral smooth contrast enhancement
								Facial NCS: Absent	
13	25	Negative	BFP	IVIG	11 days	3	N\A	Sensory NCS: UL: absent SNAPs LL: normal Motor NCS: UL and LL: Prolonged DMLs, and F‐wave latencies Slow CV Dispersed CMAPs and CB	Normal except for Bilateral smooth contrast enhancement
								Facial NCS: Absent	
13	26	Negative	BFP	IVIG		3	N\A	Sensory NCS: UL: absent SNAPs LL: normal Motor NCS: UL and LL: Prolonged DMLs, and F‐wave latencies Slow CV Dispersed CMAPs and CB, Facial NCS: Absent	Normal except for Bilateral smooth contrast enhancement
					7 days				
13	27	Negative	BFP	IVIG	12 days	3	N\A	Sensory NCS: UL: absent SNAPs LL: normal, Motor NCS: UL and LL: Prolonged DMLs, and F‐wave latencies Slow CV Dispersed CMAPs and CB, Facial NCS: Absent	Normal except for Bilateral smooth contrast enhancement
13	28	Negative		N/A	8 days	3	N\A	Sensory NCS: UL: absent SNAPs LL: normal, Motor NCS: UL and LL: Prolonged DMLs, and F‐wave latencies Slow CV Dispersed CMAPs and CB, Facial NCS: Absent	
14	29	Negative	BFP	IVIG	10 Days	1	8	Not available	Not available
15	100	Negative	N\A	N\A	42 days	N\A	N\A	Not available	Not available

Based on a patient's clinical presentation on Day 7 after admission, the modified Erasmus GBS outcome score (mEGOS) is regarded as a crucial prognostic sign that aids in predicting the long‐term fate of the patient. Therefore, the likelihood that a patient will not be able to walk independently six months after admission increases with increasing mEGOS score. The mean and standard deviation of mEGOS scores were (6 ± 5.51) and (10.38 ± 1.06) in AIDPs and non‐AIDPs, respectively(Table 4).^14^

The CSF protein level was elevated in most cases, which is a critical biomarker determining severity and extent of disease.^34^ In the AIDP group, there were two cases of albumino‐cytological dissociation, whereas in the non‐AIDP group, there were 15 cases. One patient in the AIDP group required mechanical ventilation, whereas five patients in the non‐AIDP group required mechanical ventilation (Table 4).

Acute GBS presents with proximal and distal weakness as well as significant neck flexion weakness requiring immediate intubation. GBS may also cause areflexia or hyporeflexia. Some patients may also develop dysphagia, facial diplegia, or cranial nerve involvement.^15^ Some patients developed general muscle weakness, upper limb weakness, lower limb weakness, quadriparesis, paraplegia, ascending paralysis, numbness, diarrhea, Bell's palsy, and dysphagia (Table 2). On the other hand, some patients also had bilateral facial weakness, paresthesia, quadriplegia, headache, visual disturbances, back pain, and diplopia. Patients with AIDP variants and non‐AIDP variants had numbness in 6 cases and 14 cases, respectively. Nine patients showed a state of Cranial Nerve VII palsy in our review, one of AIDP variant and eight of none AIDP, respectively (Table 4).

Bell's palsy, an autoimmune demyelinating cranial neuritis, is considered to be a mononeuritic variant of GBS, which causes the immune system to attack peripheral nerve myelin antigens.^35^ One of the AIDP variants and three non‐AIDP GBS patients both presented with Bell's palsy, in which the earliest symptoms include weakness and tingling in the extremities that quickly spread and paralyze the whole body.^13^, ^14^

GBS, characterized by chronic weakness and absent or decreased myotatic reflex, is a group of neuropathic disorders.^12^ In patients with the AIDP variant there were five upper limb weakness cases and seven lower limb weakness cases. In contrast, in patients with the non‐AIDP variant there were seven upper limb weakness cases and ten lower limb weakness cases.

The latency period between the administration of the vaccine and the appearance of GBS symptoms was 14.41 ± 7.23 days (Table 4). In reality, the delay between the injection of COVID‐19 vaccinations and the onset of GBS symptoms offers a hint to the etiology of GBS, which may be explained by the immunological reaction to the COVID‐19 vaccines resulting in peripheral nerve injury.^36^ Autoantibodies may develop against certain viral components of vaccines that cross‐react with peripheral nerves due to molecular mimicry, resulting in immune‐mediated damage to the peripheral nervous system leading to GBS.^37^

In 28 cases, the PCR test was negative, while in one, it was positive, indicating that the vaccine against SARS‐CoV‐2 caused GBS, not the COVID‐19 disease (Table 5).^38^ It is also possible to develop GBS without infection and be negative in RT‐PCR, indicating that immune‐mediated mechanisms trigger the release of PNS antigens that damage peripheral nerves.^39^

The most common treatment was intravenous immunoglobulin, or IVIG, and methylprednisolone for cases 6 and 7. (Table 5), In our cohort, we identified 17 cases in which MRI imaging was performed on the brain. Among these, 7 cases had normal findings, other cases had abnormal findings, and 22 cases had NCS findings (Table 5).

## 5 LIMITATIONS

Our study is one of the first to compare the clinical presentation, management, and outcomes of vaccinated patients who developed GBS, highlighting differences between GBS variants. In addition, we concentrated on Brighton categorization and mEGOS GBS functional rating. It is important to keep in mind various restrictions while evaluating our research. Despite our thorough search, which we believe is sufficient to capture all pertinent case series and reports, the first limitation is that there must be case reports and case series of GBS following COVID‐19 vaccine administration. As a result, there is a chance that we will miss out on new upcoming studies. Another limitation is that the patient in study number 11 had GBS after taking the vaccine and catching COVID‐19 despite being vaccinated, and we included this study as GBS cause is unclear and not confirmed regarding COVID‐19 disease or vaccination.^27^ Finally, The last included study reported by FDA was not included in the meta‐analysis because of the lack of information about 100 patients who were reported to have GBS after the COVID‐19 vaccine because there is a disproportionate number of atypical cases of GBS.^31^

## 6 CONCLUSION

In this systematic review, we examined the neurological outcomes and presentations related to the COVID‐19 vaccination. To our knowledge, this is the updated study that describes the neurological outcomes associated with GBS after the COVID‐19 vaccine. In our review, we included 14 studies with a total of 29 cases, 59% of which were males and 41% were females. Following vaccination, GBS typically appeared 14.4 days later. Most cases developed GBS after receiving the AstraZeneca/Oxford vaccine. Most patients who developed GBS following COVID‐19 vaccinations were treated with intravenous immunoglobulins. A large percentage of those included in the study have recovered or are recovering. Our systematic review has concluded that more research is needed to understand the effects of COVID‐19 vaccination on the body and its role in the development of GBS.

## AUTHOR CONTRIBUTIONS

**Nour Shaheen:** Writing – original draft; writing – review and editing. **Abdelraouf Ramadan:** Writing – original draft; writing – review and editing. **Abdulqadir J. Nashwan:** Writing – original draft; writing – review and editing. **Ahmed Shaheen:** Writing – original draft; writing – review and editing. **Shahzaib Ahmad:** Writing – original draft; writing – review and editing. **Karam R. Motawea:** Writing – original draft; writing – review and editing. **Salaheldin Mohamed:** Writing – original draft; writing – review and editing. **Rahma Mohamed Sameh:** Writing – original draft; writing – review and editing. **Sarya Swed:** Writing – original draft; writing – review and editing. **Hani Aiash:** Writing – original draft; writing – review and editing.

## FUNDING INFORMATION

The authors declare that no funding support was received for this study.

## CONFLICT OF INTEREST STATEMENT

The authors declare that they have no competing interests.

## ETHICS STATEMENT

Not applicable.

## CONSENT

Written informed consent was obtained from the patient to publish this report in accordance with the journal's patient consent policy.

## Data Availability

All data generated during this study are included in this published article.
